# A Table-Based Random Sampling Simulation for Bioluminescence Tomography

**DOI:** 10.1155/IJBI/2006/83820

**Published:** 2006-11-09

**Authors:** Xiaomeng Zhang, Jing Bai

**Affiliations:** Department of Biomedical Engineering, Tsinghua University, Beijing 100084, China

## Abstract

As a popular simulation of photon propagation in turbid media, the main problem of Monte Carlo (MC) method is its cumbersome computation. In this work a table-based random sampling simulation (TBRS) is proposed. The key idea of TBRS is to simplify multisteps of scattering to a single-step process, through randomly table querying, thus greatly reducing the computing complexity of the conventional MC algorithm and expediting the computation. The TBRS simulation is a fast algorithm of the conventional MC simulation of photon propagation. It retained the merits of flexibility and accuracy of conventional MC method and adapted well to complex geometric media and various source shapes. Both MC simulations were conducted in a homogeneous medium in our work. Also, we present a reconstructing approach to estimate the position of the fluorescent source based on the trial-and-error theory as a validation of the TBRS algorithm. Good agreement is found between the conventional MC simulation and the TBRS simulation.


Hindawi Publishing Corporation
International Journal of Biomedical Imaging
Volume 2006, Article ID 83820, Pages 1–8
DOI 10.1155/IJBI/2006/83820

### A Table-Based Random Sampling Simulation for Bioluminescence Tomography

**Xiaomeng Zhang and Jing Bai**

*Department of Biomedical Engineering, Tsinghua University, Beijing 100084, China*

Received 16 February 2006; Revised 20 May 2006; Accepted 13 August 2006

Recommended for Publication by Jie Tian

As a popular simulation of photon propagation in turbid media, the main problem of Monte Carlo (MC) method is its cumbersome computation. In this work a table-based random sampling simulation (TBRS) is proposed. The key idea of TBRS is to simplify multisteps of scattering to a single-step process, through randomly table querying, thus greatly reducing the computing complexity of the conventional MC algorithm and expediting the computation. The TBRS simulation is a fast algorithm of the conventional MC simulation of photon propagation. It retained the merits of flexibility and accuracy of conventional MC method and adapted well to complex geometric media and various source shapes. Both MC simulations were conducted in a homogeneous medium in our work. Also, we present a reconstructing approach to estimate the position of the fluorescent source based on the trial-and-error theory as a validation of the TBRS algorithm. Good agreement is found between the conventional MC simulation and the TBRS simulation.

Copyright © 2006 X. Zhang and J. Bai. This is an open access article distributed under the Creative Commons Attribution License, which permits unrestricted use, distribution, and reproduction in any medium, provided the original work is properly cited.

### 1. INTRODUCTION

The study of light propagation in a highly scattering or turbid medium has attracted growing interest among researchers around the world because of the potential applications to medical problems, such as imaging. With the noninvasive nature, the recent developed bioluminescence optical tomography (BLT) soon became a hotspot in optical imaging and shed light on the early detection of pathological changes of biological tissues. However, there are still some obstacles preventing the realization of this technique, among which the most serious problem is the strong scattering in biological tissues. In most published works, a computable model of the propagation of radiation in tissue, most often the diffusion approximation, is adopted as appropriate for cases that are scattering-dominated, that is, where $\mu_s \gg \mu_a$. However, an analytical and direct solution to the diffusion equation or the Boltzmann transport equation is not practically affordable due to the computational complexity, except in extremely simplified situation [1, 2].

Therefore, numerical simulation plays a critical role in BLT, among which the Monte Carlo (MC) approach is important for its accuracy and flexibility. Okada et al. used the MC method to describe the spatial distribution of photon paths [3]. Li et al. portrayed a simulation for bioluminescent tomography with MC approach [4]. Despite that most existing MC programs are based on a geometric difference from the biological tissues [5–10], the main problem of these works is the cumbersome and time-consuming computations. Therefore, reducing the computing time of MC method is a crucial problem for further study.

In this paper, we present table-based random sampling (TBRS) simulation to accelerate the computation, thus enhance the efficiency of conventional MC simulation. In Section 2.1, a conventional MC algorithm for BLT was described for accurately simulating the whole process of photons and obtaining physical quantities, also laying basis to the TBRS algorithm. In Section 2.2, the TBRS algorithm was described in detail. Section 3 presents a reconstruction example of the fluorescent source with trial-and-error method as a verification of the TBRS algorithm. Then in Sections 4 and 5 and *Discussion*, we provide results of conventional MC simulation and TBRS simulation, as well as their comparisons.

### 2. METHODOLOGY

### 2.1. Conventional MC simulation

The conventional MC method is based on randomly constructing a set of trajectories [11], which mimic the real photon migration process in tissue. The photon propagation in biological tissues includes the optical process of absorption, scattering, reflection, and transmission. In a dominantly scattering medium, the azimuthal angle $\varphi$ and deflection angle $\theta$ of moving photons should be obtained from MC random sampling, in which $\varphi$ is uniformly distributed over the interval $(0, 2\pi)$, and $\cos\theta$ is described by the Henyey-Greenstein function [12]:

$$f(\cos\theta) = \frac{1 - g^2}{2\left(1 + g^2 - 2g\cos\theta\right)^{3/2}}. \quad (1)$$

Here, geometric parameters include the coordinates and shapes of biological tissues. The Cartesian coordinate system and a moving spherical system are used with the same origin. The azimuthal angle $\varphi$ and deflection angle $\theta$ are the two basic parameters in this spherical system. The parameter $g$ is called anisotropy factor. For biological tissues, the factor $g$ is rather close to 1, which corresponds to the fact that the deflection angle tends to be very small.

Once the microscopic scattering model has been established, conventional MC methods launch random trajectories using relevant probability densities. There is no other restriction on the simulation process. Now taking a photon's transportation for example, the position of the photon is represented by Cartesian coordinates $(x, y, z)$. The direction of photon propagation is represented by the directional cosines $(\mu_x, \mu_y, \mu_z)$:

$$\begin{aligned} \mu_x &= \sin\theta\cos\varphi, \\ \mu_y &= \sin\theta\sin\varphi, \\ \mu_z &= \cos\theta. \end{aligned} \quad (2)$$

Here, $\cos\theta = 2\xi_\theta - 1$ and $\varphi = 2\pi\xi_\varphi$, $\xi_\theta$ and $\xi_\varphi$ are uniform random numbers in (0,1).

The photon has an initial energy of $w$ (termed weight). When the photon launches from a light source, positional sampling finds the initial position of the photon, while angular sampling decides the direction of photon transportation. Then the step size $\lambda$, which is a random variable, determines the next interaction site of this photon. Also it should be modified if the photon hits the boundary of the ambient medium. After each scattering, the energy of the photon will partly be absorbed by medium/tissues. Both $\lambda$ and weight loss can be calculated based on the absorption coefficient $\mu_a$, the scattering coefficient $\mu_s$, and the anisotropy factor $g$. Finally, the photon will reach the boundary of the medium/tissues and may either be reflected or transmitted. According to the Snell discipline and Fresnel formula,

$$n_i \sin\alpha_i = n_t \sin\alpha_t, \quad (3)$$

where $\alpha_i$ is the angle of incidence, $\alpha_t$ is the angle of transmission, $n_i$ and $n_t$ are refractive indices of the media where the photon incidents from and transmits to. Assuming that $\alpha_i$ is very small, we can obtain a simplified expression of the internal reflectance $R(\alpha_i)$:

$$R(\alpha_i) = \frac{(n_2 - n_1)^2}{(n_2 + n_1)^2}. \quad (4)$$

Therefore, whether the photon will transmit the boundary or be internally reflected by the boundary can be determined.

In this paper, both conventional MC simulation and the TBRS simulation are conducted in a homogeneous medium. We use a cylinder-shape container to simulate biological tissues like a mouse's chest. There are 24 sensors on the side of the cylinder, as shown in Figure 1, the sensors are uniformly arranged in three layers. The conventional way to simulate the input source in a diffusion model is to represent the source by a diffuse point source located at $r$ in the medium. The photon that first launched at $r$ will travel through the medium until it exits the boundaries of the medium. Only a small part of photons will be received by sensors, so a large number of photons should be used to make the simulation results statistically reliable. Yet, the conventional MC model is fairly time consuming. Thus, an improved MC algorithm, termed as TBRS simulation, was developed to increase the efficiency. Also, with the data acquired in the simulation, we estimated the location of the source.

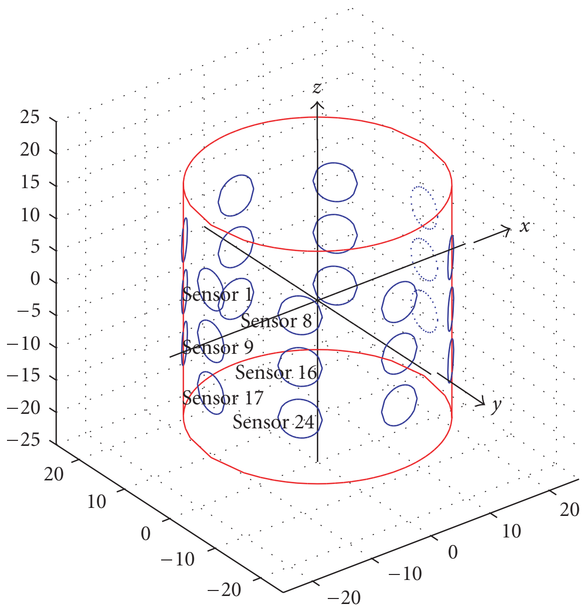

Figure 1: Diagram of the simulated cylinder medium with fluorescent source and 24 detectors. The center of cylinder was set to coordinates (0, 0, 0). The cylinder has a height of 36 mm and 18 mm as base radius. Each detector has a radius of 3 mm and centers of the sensors are on the $z$-plane of $z = 11$, $z = 0$, and $z = -11$, respectively. Sensors in three layers are numbered clockwisely (1–8, 9–16, 17–24). The fluorescent source (•) was set in $r$ ($r$ can be any location in the cylinder in our simulation).

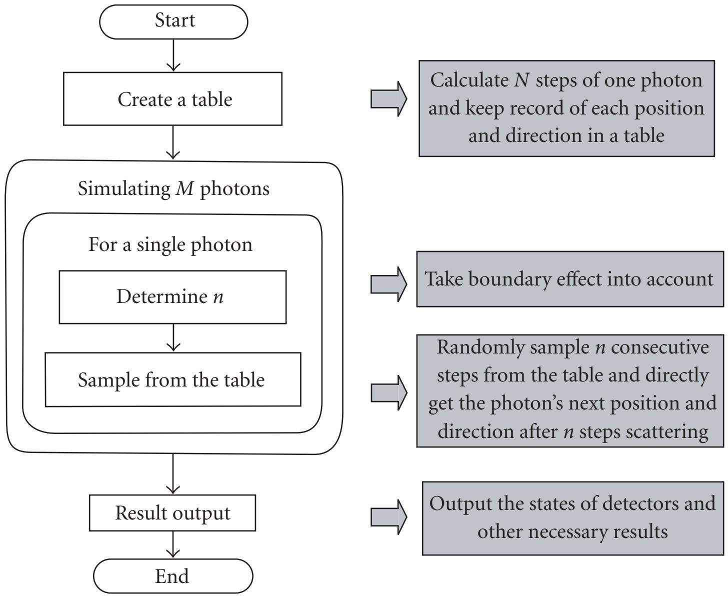

Figure 2: The flowchart to show the algorithms of TBRS method. Assuming a single-photon transport $N$ steps from the initial position, we calculate and record the position and direction of each step in a table. $M$ is the total number of photons used in a simulation.

### *2.2. TBRS simulation algorithm*

In the conventional MC method, a single-step iterative function composed of the formulae derived from H-G function [12] should be created. We make an iterative operation for each step movement of one photon to get the next position and direction of it. Each photon may transport hundreds of steps before going across the boundary or being received by detectors. A large number of photons were used in a simulation, so most of the computing time is used in photon scattering. As described in Section 2.1, when a photon transports from one site to another in one step, the direction and step size are both random variables generated from the iterative function. Because the iterative function in the conventional MC algorithm has already used a set of algebraic equations instead of the diffusion equation to make the iteration simple, rather than changing the function, we alternatively consider expediting the simulation by reducing iteration times.

The key to our improved algorithm is to simplify multisteps scattering to a single-step process. Now assume that the photon is in one site, then, after $n$ steps, its position and scattering direction become two random variables which have certain distributions determined by the single-step iterative function. To obtain such a distribution quickly, a table-based random sampling method was proposed as shown in Figure 2. At the beginning, we assume that a photon is in the initial position. Then we simulate $N$ steps of its transportation in a boundless reference medium with conventional MC method. The results obtained form a table which contains the position and direction of the photon in each step. All the $N$ positions and directions are listed in order from the initial step to the last. For any consecutive $n$ ($n \ll N$) steps in the $N$ steps of photon movement, they suggest a possible state of continuous-$n$-step transportation. There are in sum $(10^7 - n + 1)$ different states containing enough possibilities of the continuous-$n$-step transportation of one photon. Here we will introduce an $n$-calculation. Now assume a photon is in site 1, its position and direction after $n$ steps are randomly distributed. What we do is to randomly take out continuous $n$ steps from the table to mimic the photon's movement. This is defined as a random sampling process in the TBRS method. With the $n$ steps taken from the table, the change of position and direction (denoted by coordinates $x$, $y$, $z$, and $\varphi$, $\theta$) from the initial to the end within these $n$ steps is obtained. Then this change is added to the position and direction of simulating photon in site 1, thus the position and direction of this photon after $n$ steps, which we define as site 2, can be directly calculated. Therefore, the calculation of $n$ separate steps of photon transportation is replaced by just querying the table once, regardless of the position and direction of the photon where we start the $n$-calculation. Once the position and direction of the photon in site 2 are obtained, the $n$-calculation described above will be repeated to obtain site 3, site 4, and so forth. Theoretically, unless the photon crosses the media boundary during the $n$ steps, this algorithm can be applied to calculate the next position of any photon transporting $n$ steps from one position. Real simulation has showed that TBRS method can greatly increase the computing speed to several times of that of the conventional MC method in different applications.

Then we discuss how to determine $N$, the size of the table. The larger the $N$ value is, the lower repetition rate of the data stored in that table is. See Figure 3, when $N$ ranges from $10^2$ to $10^7$, we take out some $N$ values $(100, 200, 300, \ldots, 1000, 2000, \ldots, 1000000, 2000000, \ldots, 10^7)$. For each $N$, we simulate $10^7$ photons using the TBRS method (assume

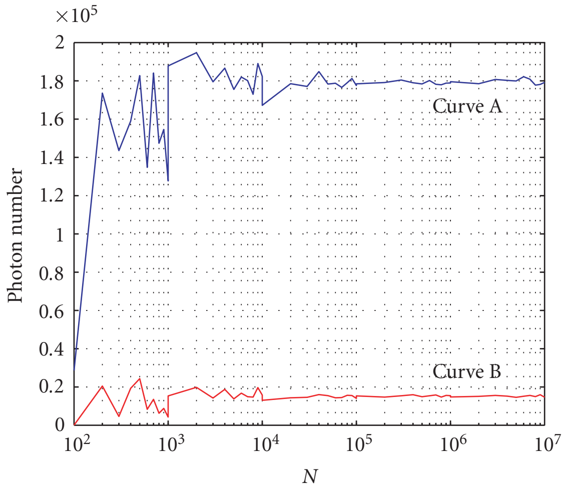

Figure 3: TBRS simulation results with different table sizes. $N$ denotes the table size. Curve A and Curve B denote the maximized and minimized numbers of photons received among the 24 sensors in the simulations.

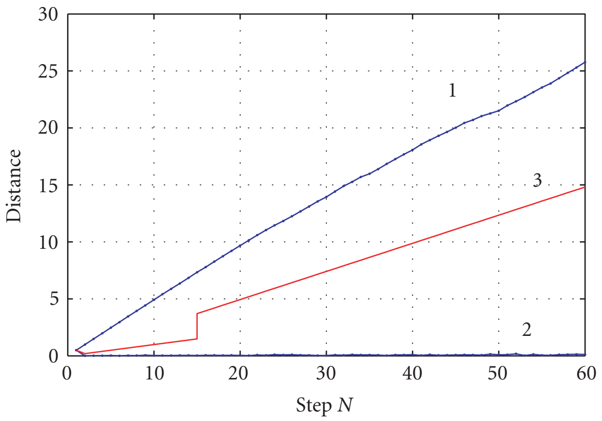

Figure 4: An experimental way to determine $n$: the relationships between $D$ and $n$ in different conditions. $n$ denotes the number of steps a photon travels, and $D$ denotes the distance between the position of the photon and the nearest media boundary. The range of a single step is constrained to 5, so line 1 begins at $D = 5$.

the source is in $(3, -4, 5)$, in the phantom shown in Figure 1). In each simulation, the maximized and minimized numbers of photons received among the 24 detectors can be derived, forming Curve A and Curve B. Apparently, when $N$ ranges from $10^2$ to $10^4$, both Curve A and Curve B fluctuate greatly. When $N$ becomes larger, the curves go stable. It shows that as far as $N$ is larger than a certain value (here is $10^4$), the simulation results become stable, with little influence from the size of the table. This dividing value is experimentally determined, as shown in Figure 3. Be aware that the dividing values vary according to different simulation environments (phantom properties, optical properties, etc). We should use the experimental method explained in this paragraph to determine those values. Theoretically, the table size $N$ can be any value that is greater than the dividing value.

The value of $n$ is another important factor to influence the effectiveness of the TBRS algorithm. Ideally we want to maximize $n$ to accelerate the computing as much as possible, but the prerequisite is that the photon will not cross the boundary during the $n$ steps of scattering. Now we discuss how to take the boundary into account by selecting an appropriate $n$. Assume that the scattering photon is now in one position, and the distance between this position and the nearest media boundary is $D$. To make sure there will be no cross-bound phenomenon during the next $n$ steps, the most direct way to determine $n$ is using $n = D/\lambda$. Therefore, $n$ is a constantly changing variable. Every time we determine a photon's position and direction after $n$ steps by randomly sampling the table, we should first determine an optimal $n$ with this formula. Then the time of $n$ scatterings in the conventional MC simulation can be replaced by the time of querying the table, which is much less, relatively.

However, is there a better way to determine $n$, an optimal $n$ that is as large as possible to achieve better efficiency of the TBRS algorithm? In fact, the simulation results using $n = D/\lambda$ are good enough to prove the superiority of the TBRS algorithm. Yet experiential data can help to slightly improve the efficiency of our TBRS method by getting a more optimal $n$. In the established table, the distance of randomly distributed any continuous $n$ steps can be obtained ($n = 1, 2, \ldots, N$). For $n = 1, 2, \ldots, N$, there will be maximized distance and mean distance for each $n$ steps. In Figure 4, Line 1 represents the relationship between $D$ and $n$ when $n$ assuming the photon attains maximized distance for every $n$ steps, thus the space below Line 1 will be "safe" with no cross-bound phenomenon. Line 2 represents the relationship between $D$ and $n$ when $n$ assuming the photon attains minimized distance for every $n$ steps. Here, Curve 2 seems to be much smoother than Curve 1. In fact, both Curve 1 and Curve 2 are formed by discrete values. But for Curve 2, when $n$ increases, the distance increment is much smaller, making the curve very "continual." Then consider $n$ as a variable of $D$ in real simulation, say $n = F(D)$. In order to avoid cross-bound phenomena and attain higher efficiency, the function $F(\cdot)$ is set as illustrated by Line 3. The lower part of Line 3 is close to the Line 2 to maximize $n$, and the higher part of Line 3 is close to Line 1 to avoid any cross-bound phenomenon. Line 3 is only an experiential curve based on some preliminary simulation results, which can surely be replaced by some alternatives. However, any attempt to increase $n$ is at the risk of losing accuracy of the simulation results. TBRS is a validating numerical method, in which $n$ should be appropriately determined with the assurance of its accuracy.

### 3. RECONSTRUCTION

The studies of inverse problems of bioluminescence optical diffusion include the reconstruction of optical parameters and reconstruction of fluorescent sources. Our work is sorted to the latter. In this work, reconstruction of fluorescent source served as a verification of the feasibility of our

TBRS algorithm. Here the key to the reconstruction is the concept of trial and error. A TBRS simulation is performed prior to reconstruction. With the numbers of photons received by all the 24 detectors obtained from the simulation as input states, detailed information about the simulation environment (including the shape, size, and optical parameters of the medium), size, and shape of detectors shown in Figure 1, we can start the reconstruction process and estimate the position of sources in the 3D medium with fine precision.

A stepwise searching was used for source location estimation. We set the origin $(0, 0, 0)$ in the Cartesian system as the initial searching position. Then, we do several TBRS simulations of 1000 to 50000 photons that launched this position. Comparing the input states with the data acquired in this TBRS simulation, we can get the next modified searching position through a set of calculations. Then the calculations are performed again and again to get new searching positions. Use $(x, y, z)$ to denote the searching position, the increments in $x$, $y$ direction can be calculated by the following formulae:

$$\begin{gathered}(dx)_i = R \times \cos(\varphi_i) - x, \\ (dy)_i = R \times \sin(\varphi_i) - y, \\ \Delta x = x\,\text{step} \times \sum_{1}^{24} \left( \frac{(dx)_i}{\sqrt{(dx)_i^2 + (dy)_i^2}} \right) \times t_{xy}^{(\Delta \det_i)}, \\ \Delta y = x\,\text{step} \times \sum_{1}^{24} \left( \frac{(dy)_i}{\sqrt{(dx)_i^2 + (dy)_i^2}} \right) \times t_{xy}^{(\Delta \det_i)},\end{gathered} \tag{5}$$

where $\varphi_i$ is the rotation angle of the center of $i$th detector, $i = 1, 2, \ldots, 24$. $x$ step and $t_{xy}$ are both position increments constant in the $x$, $y$ direction. $\Delta \det_i$ denotes the difference between the normalized input number of photons of the $i$th detector and the normalized number of photons received by this detector in one searching, $i = 1, 2, \ldots, 24$. Here "normalized" means *number of photons received by the* $i$th *detector/the total number of photons*. In our reconstruction, $x\,\text{step} = 1$, $t_{xy} = 7.5$.

The increment of $z(\Delta z)$ is

$$\begin{aligned}\Delta z_s &= \left( \frac{\text{Sum}_{\text{up}}}{\text{Sum}_{\text{mid}}} + \frac{\text{Sum}_{\text{up}}}{\text{Sum}_{\text{down}}} \right) \\ &\quad - \left( \frac{\text{Sum}_{\text{down}}}{\text{Sum}_{\text{mid}}} + \frac{\text{Sum}_{\text{down}}}{\text{Sum}_{\text{up}}} \right), \\ \Delta z_r &= \left( \frac{\text{Sum}^r_{\text{up}}}{\text{Sum}^r_{\text{mid}}} + \frac{\text{Sum}^r_{\text{up}}}{\text{Sum}^r_{\text{down}}} \right) \\ &\quad - \left( \frac{\text{Sum}^r_{\text{down}}}{\text{Sum}^r_{\text{mid}}} + \frac{\text{Sum}^r_{\text{down}}}{\text{Sum}^r_{\text{up}}} \right), \\ \Delta z &= t_z \times (\Delta z_r - \Delta z_s),\end{aligned} \tag{6}$$

where $\text{Sum}_{\text{up}}$, $\text{Sum}_{\text{mid}}$, and $\text{Sum}_{\text{down}}$ denote the total photons received by the detectors of the upper layer, the middle layer, and the lower layer in the input states, while $\text{Sum}^{\gamma}_{\text{up}}$, $\text{Sum}^{\gamma}_{\text{mid}}$, and $\text{Sum}^{\gamma}_{\text{down}}$ denote the photons received by each layer of detectors in one searching. $t_z$ is the increment constant in $z$ direction, which is used to adjust the step size of searching. After a number of searches, a favorable position of source with which the simulation results are closest to the input states will be obtained, based on a given limit of error.

### 4. RESULTS

Our implementation was based on the C++ language, so the computation speed is generally tolerable.

### *Comparison between the conventional MC simulation and the TBRS simulation*

Various phantom and in vivo experiments [4, 13] have verified that the conventional MC method is capable of providing accurate predictions of photon propagation in turbid medium. We can therefore compare their results with outcomes from the TBRS method, in an attempt to verify our algorithm. In both MC simulations, the number of photons we used is $10^7$, the absorption coefficient and reduced scattering coefficient are $0.025\,\text{mm}^{-1}$ and $2\,\text{mm}^{-1}$, and the anisotropy factor $g$ is 0.8. Figures 4 and 5 illustrate the comparison between the conventional MC simulation and the TBRS simulation when the fluorescent source was, respectively, put in $(0, 0, 0)$ and $(5, -6, 9)$. Along the $x$-axis, coordinate $i$ denotes the $i$th detector, $i$=1, 2, ..., 24. In both Figures 6 and 7, $y$ coordinate denotes the number of photons. As shown in Figures 6 and 7, the results generated by these two MC simulations were very close to each other. We also recorded computing time in both simulations. On an IBM compatible PC with 1.86 GHz CPU, 1 G RAM, and Windows XP operating system, the average running time of the TBRS simulation was 1183 seconds with source in $(0, 0, 0)$, 1267 seconds with source in $(5, -6, 9)$, while it costs 3272 seconds and 3266 seconds by the conventional MC simulation, respectively.

### *The influence of phantom size*

Because the sampling length $n$ relates to the distance between the photon and the media boundary, when the phantom size becomes larger, the superiority of the TBRS algorithm becomes greater. Figure 8 represents the influence of phantom size on the simulation time. The curve depicts the change of simulation time when $R$ changes from 15 to 60. $R$ is the radius of the cylinder, which is equal to half the height of the cylinder.

### *Estimation of source location*

With the number of photons received by each of the 24 sensors from TBRS simulation, the location of the fluorescent source can be estimated. Assume that the fluorescent source was in $(5, -6, 9)$ in the TBRS simulation, the stepwise

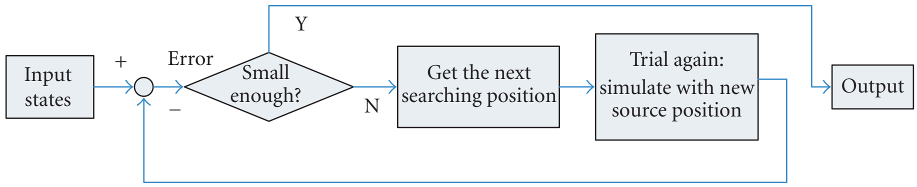

FIGURE 5: Algorithmic structure used for reconstruction.

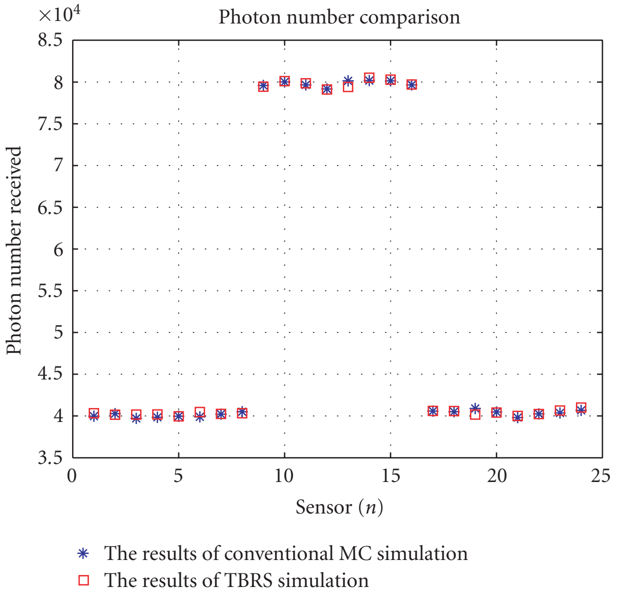

FIGURE 6: Photons received by the 24 sensors with source in (0, 0, 0).

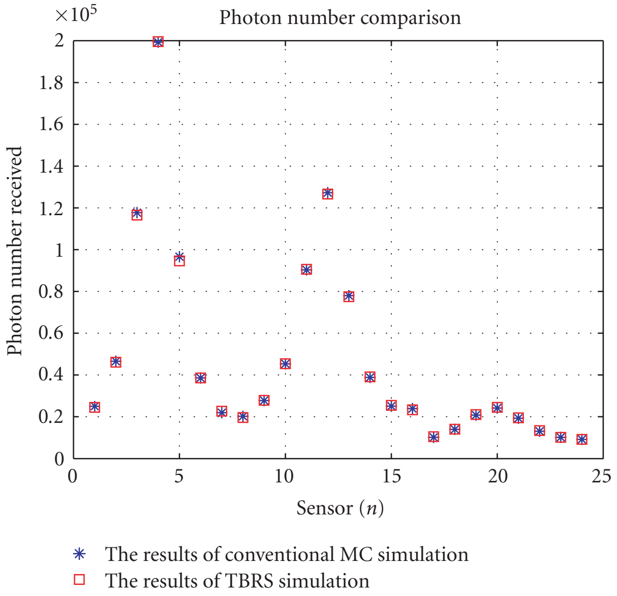

FIGURE 7: Photons received by the 24 detectors with source in (5, −6, 9).

searching process is shown in Figure 9. Once the error is below 0.05, the searching process is terminated. The estimated source location was (4.969554, −5.910204, 8.807371).

### 5. DISCUSSION

In Figures 6 and 7, as can be seen, there are good agreements between the results with the conventional MC algorithm and the TBRS algorithm for different locations of the fluorescent source. Meanwhile, the average running time of the TBRS simulation is much less than the conventional MC simulation. Simulations with different geometric media and fluorescent sources were conducted with the TBRS, and we still obtained results in accordance with those of the conventional MC simulation. Thus, the TBRS algorithm will significantly increase efficiency compared to the conventional MC method. The randomicity of TBRS is guaranteed by selecting appropriate $N$, $n$, and have a reliable mechanism to generate random numerical values.

The superiority of TBRS algorithm, theoretically, is the reduced computing complexity. Further work will focus on simplifying the computing process in this algorithm to get quantum reduction of the simulation time, with the maintenance of accuracy. Using a table still requires some mathematical calculation work, which has a negative effect on the efficiency of TBRS. So efforts are needed to simplify some parts of the TBRS.

There are other questions which might be raised, for example as follows. Is the TBRS still useful in a heterogeneous media with intermedium boundary? The answer is positive. A merit of the TBRS algorithm is that it can adapt to different geometric mediums, except that the determination of $n$ becomes more complicated in heterogeneous media, for there is a higher occurrence of cross-bound phenomena during $n$ scatterings of one photon with more existing boundaries. It is possible that $n$ will be restrained to a smaller value so that the efficiency of TBRS may suffer a tiny decrease. Yet generally, the potential applications of TBRS in heterogeneous media/biological tissues are promising. In our further work, simulations and phantoms will be conducted to verify this assertion. Reconstruction of the fluorescent source, in this work, is a validation of the simulation results of the TBRS algorithm. We used a method derived from trial-and-error theory, which was easy to understand and implement. The disadvantage of this method was that it required a priori knowledge of the optical properties of reference media. Further improvement or other useful algorithms need to be proposed to solve the problem.

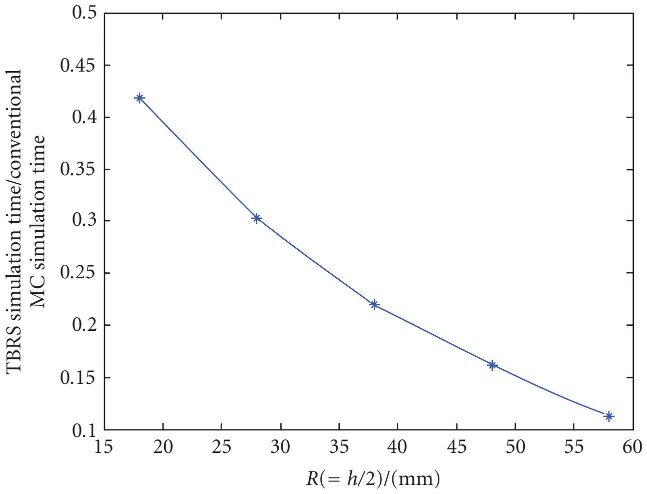

FIGURE 8: How the phantom size influences the simulation time.

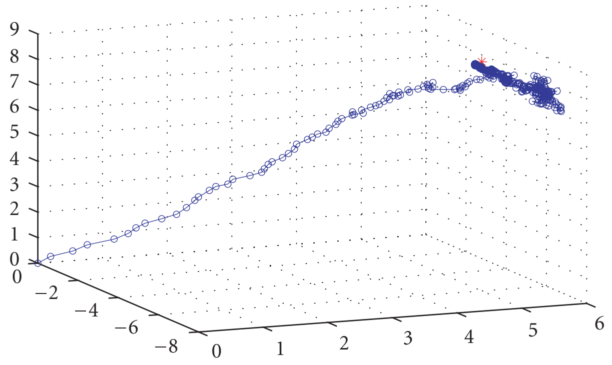

FIGURE 9: The path of searching for the favorable source position.

### ACKNOWLEDGMENTS

The work in hand was partially supported by NNSFC and NBRPC2006CB705700.

### REFERENCES

[1] I. V. Yaroslavsky, H.-J. Schwarzmaier, A. N. Yaroslavsky, and V. V. Tuchin, "Radiative transfer equation and its diffusion approximation in the frequency domain technique: a comparison," in *Photon Transport in Highly Scattering Tissue*, vol. 2326 of *Proceedings of SPIE*, pp. 465–474, Lille, France, September 1995.

[2] A. X. Cong and G. Wang, "Multispectral bioluminescence tomography: methodology and simulation," *International Journal of Biomedical Imaging*, vol. 2006, Article ID 57614, 7 pages, 2006.

[3] E. Okada, M. Firbank, and D. T. Delpy, "The effect of overlying tissue on the spatial sensitivity profile of near-infrared spectroscopy," *Physics in Medicine and Biology*, vol. 40, no. 12, pp. 2093–2108, 1995.

[4] H. Li, J. Tian, F. Zhu, et al., "A mouse optical simulation environment (MOSE) to investigate bioluminescent phenomena in the living mouse with the Monte Carlo method," *Academic Radiology*, vol. 11, no. 9, pp. 1029–1038, 2004.

[5] L. Wang, S. L. Jacques, and L. Zheng, "MCML—Monte Carlo modeling of light transport in multi-layered tissues," *Computer Methods and Programs in Biomedicine*, vol. 47, no. 2, pp. 131–146, 1995.

[6] A. D. Kim and J. B. Keller, "Light propagation in biological tissue," *Journal of the Optical Society of America A*, vol. 20, no. 1, pp. 92–98, 2003.

[7] S. T. Flock, M. S. Patterson, B. C. Wilson, and D. R. Wyman, "Monte Carlo modeling of light propagation in highly scattering tissues - I: model predictions and comparison with diffusion theory," *IEEE Transactions on Biomedical Engineering*, vol. 36, no. 12, pp. 1162–1168, 1989.

[8] S. T. Flock, B. C. Wilson, and M. S. Patterson, "Monte Carlo modeling of light propagation in highly scattering tissues - II: comparison with measurements in phantoms," *IEEE Transactions on Biomedical Engineering*, vol. 36, no. 12, pp. 1169–1173, 1989.

[9] S. R. Arridge, M. Schweiger, M. Hiraoka, and D. T. Delpy, "A finite element approach for modeling photon transport in tissue," *Medical Physics*, vol. 20, no. 2, pp. 299–310, 1993.

[10] S. A. Prahl, M. Keijzer, S. L. Jacques, and A. J. Welch, "A Monte Carlo model of light propagation in tissue," in *Dosimetry of Laser Radiation in Medicine and Biology*, vol. IS 5 of *Proceedings of SPIE*, pp. 102–111, Bellingham, Wash, USA, 1989.

[11] N. G. Chen and J. Bai, "Estimation of quasi-straightforward propagating light in tissues," *Physics in Medicine and Biology*, vol. 44, no. 7, pp. 1669–1676, 1999.

[12] L. G. Henyey and J. L. Greenstein, "Diffuse radiation in the galaxy," *Astrophysical Journal*, vol. 93, pp. 70–83, 1941.

[13] A. Sassaroli, C. Blumetti, F. Martelli, et al., "Monte Carlo procedure for investigating light propagation and imaging of highly scattering media," *Applied Optics*, vol. 37, no. 31, pp. 7392–7400, 1998.

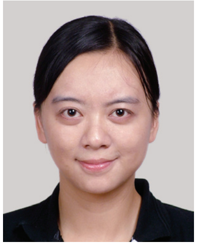

**Xiaomeng Zhang** received the B.S. degree in biomedical engineering from the Department of Biomedical Engineering at Tsinghua University in July 2006. She will start pursuing her M.S. and Ph.D degrees in the Department of Electrical Engineering at Stanford University in September 2006. Her research interests include biomedical imaging (molecular/optical imaging, MRI, etc.), image analysis, biomedical signal processing, and bioelectrical engineering.

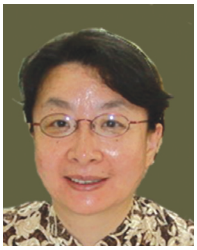

**Jing Bai** obtained the M.S. and Ph.D. degrees from Drexel University, Philadelphia, Pa, in 1983 and 1985, respectively. From 1985 to 1987, she was a Research Associate and an Assistant Professor with the Biomedical Engineering and Science Institute of Drexel University. In 1988, 1991, and 2000, she was an Associate Professor, Professor, and Cheung Kong Chair Professor, respectively, in the Department of Electrical Engineering at Tsinghua University, Beijing, China. Currently, she is with the Department of Biomedical Engineering at the same university.

